# Perplexities in Connection with Extraction of Teeth

**Published:** 1904-05

**Authors:** Henry W. Gillett


### PERPLEXITIES IN CONNECTION WITH EXTRACTION OF TEETH.[1]

BY HENRY W. GILLETT, D.M.D.

The matter of which I wish to speak first will perhaps seem to you a barren one to present before this society. Certain perplexities of my own in connection with the extraction of teeth have led me to speak of them, with the hope that I, at least, may get some benefit from your comments. I am not going to open the subject of when to extract, but will assume that most of us occasionally feel it necessary to advise the extraction of an impacted wisdom-tooth, or to insist upon the removal of useless foul and decaying roots. I also assume that most of us prefer that these operations should be performed by an "extracting specialist."

This is, at least, a fair statement of my own position, and I am confident that in the main it agrees with the practice of many of you.

My own experience in sending difficult cases of extraction to so-called extracting specialists has been unsatisfactory, in that they have always returned to me with what has seemed to me an unreasonable amount of inflammation and irritation of the parts adjacent to the operative field. Having spent much of my professional life in a small city where extracting specialists were not

[1] Read before The New York Institute of Stomatology, January 5, 1904.

accessible, I have found it necessary to do some such operations myself. The very marked difference in the sequelæ of cases where the mouth has been thoroughly sprayed immediately before and after the operation with some sterilizing agent, preferably a peroxide of hydrogen solution, as compared with similar cases where this course has not been followed, has forced itself so strongly upon my attention that I now consider such spraying an essential preliminary to the extraction of teeth. Similar spraying after the operation seems to me desirable but possibly less necessary.

The means for preventing the pain of the operation itself have been developed till it makes tooth extraction almost too attractive to the possessor of painful but still salvable teeth, but in many cases the frequently more serious suffering which follows operations in a septic field seems to be taken as a necessary evil, and no effort is made to prevent it.

It would seem as if, in many cases, the only precautions taken by the operator were to ascertain with certainty which tooth its possessor wishes to part with, and to give his anæsthetic so as to avoid pain while the operation is in actual progress.

Personally, I feel that this ruthless tearing away of an organ with little or no regard for the welfare of adjacent tissues is much too common to do us credit. What surgeon would endorse even the smallest cutting operation in any other portion of the human anatomy, when it was surrounded by removable calcular deposits, swarming with all kinds of bacteria, and bathed in fluid bearing pyogenic organisms from adjacent pus-pockets, without preliminary efforts at cleaning and sterilizing the operative field. Yet in this twentieth century this is what many self-styled "Oral Surgeons" are doing.

I realize that some of you will say it is practically impossible to sterilize the mouth. This is undoubtedly true. It is also undoubtedly true that the mouth bacteria may be so thoroughly subjugated in a few minutes as to result in great practical benefit to the patient undergoing operations at that point.

This statement has been preliminary to presenting for your consideration the question whether this society, in view of the importance to its members of knowing that their patients are to receive satisfactory attention with due regard to the progress of surgical knowledge, may assume the duty of appointing a committee to formulate the general rules which seem desirable to observe in such operations, and shall (either with or without preliminary report to the society, as may be deemed best) call to the attention of such local practitioners as advertise themselves as extracting specialists the formulated rules, and, upon invitation by such practitioners, inspect their operating-rooms with a view to reporting to the society the names of those who are found equipped for the carrying out of these rules. This at first thought may seem inquisitorial, but when it is remembered that these practitioners appeal to us for their patronage, and that there is often laxness in the essentials for common cleanliness, not to mention the requirements of asepticism in their establishments, it seems to me a warrantable inquiry, and one which those extracting specialists who realize its value to them will hasten to meet in such manner as to insure the approval of the committee.

